# Effects of Continuously Feeding Diets Containing Cereal Ergot Alkaloids on Nutrient Digestibility, Alkaloid Recovery in Feces, and Performance Traits of Ram Lambs

**DOI:** 10.3390/toxins9120405

**Published:** 2017-12-19

**Authors:** Stephanie Coufal-Majewski, Kim Stanford, Tim McAllister, Yuxi Wang, Barry Blakley, John McKinnon, Mary Lou Swift, Alexandre V. Chaves

**Affiliations:** 1 Faculty of Science, School of Life and Environmental Sciences, University of Sydney, Sydney, NSW 2006, Australia; Smaj3498@uni.sydney.edu.au (S.C.-M.); Alex.Chaves@sydney.edu.au (A.V.C.); 2 Livestock Research Section, Alberta Agriculture and Forestry, Lethbridge, AB T1J 4V6, Canada; 3 Science and Technology Branch, Agriculture and Agri-Food Canada, Lethbridge, AB T1J 4B1, Canada; Tim.McAllister@agr.gc.ca (T.M.); Yuxi.Wang@agr.gc.ca (Y.W.); 4 Department of Veterinary Biomedical Sciences, University of Saskatchewan, Saskatoon, SK S7N 5B4, Canada; brb237@mail.usask.ca; 5 Department of Animal Science, University of Saskatchewan, Saskatoon, SK S7N 5B4, Canada; john.mckinnon@usask.ca; 6 Ruminant Nutrition, Hi-Pro Feeds, Okotoks, AB T1S 1A2, Canada; MaryLou.Swift@hiprofeeds.com

**Keywords:** ergot, alkaloid, epimer, growth, lambs, nutrient digestibility

## Abstract

Allowable limits for cereal ergot alkaloids in livestock feeds are being re-examined, and the objective of this study was to compare nutrient digestibility, growth performance and carcass characteristics of ram lambs fed a range of alkaloid concentrations, including the maximum currently allowed in Canada (2 to 3 ppm). Four pelleted diets were fed: control, with no added alkaloids; 930; 1402; and 2447 ppb alkaloids based on total *R* and *S* epimers. Eight ram lambs (30.0 ± 3.1 kg) were used to examine the impacts of dietary treatments on nutrient digestibility and alkaloid recovery from feces. Concentrations of dietary alkaloids evaluated did not affect nutrient digestibility or N metabolism. Excepting ergocornine and ergocryptine, recovery of alkaloids in feces varied among periods, suggesting that individual lambs may differ in their ability to metabolize ergocristine, ergometrine, ergosine, ergotamine and their *S* epimers. In a second experiment, ram lambs (*n* = 47, 30 ± 8 kg) were randomly assigned to a diet and weighed weekly until they achieved a slaughter weight of ≥ 45 kg (average 9 weeks; range 6 to 13 weeks). Intake of DM did not differ (*p* = 0.91) among diets, although lambs fed 2447 ppb alkaloids had a lower (*p* < 0.01) ADG than did lambs receiving other treatments. The concentration of serum prolactin linearly declined (*p* < 0.01) with increasing alkaloids. Feeding 2447 ppb total alkaloids negatively impacted growth, while feeding 1402 ppb did not harm growth performance, but reduced carcass dressing percentage. Due to different concentrations of alkaloids affecting growth and carcass characteristics in the present study, determining allowable limits for total dietary alkaloids will require a better understanding of impacts of alkaloid profiles and interactions among individual alkaloids.

## 1. Introduction

Allowable limits for cereal ergot alkaloids in livestock diets are being re-examined in many parts of the world, but are complicated by a number of factors. As alkaloid concentrations of ergot bodies are extremely variable [1], existing regulations for allowable ergot bodies in grain may conflict with maximum allowable ergot alkaloid concentrations in feed. Furthermore, types of alkaloids present in ergot vary by location and cereal species [2]. To date, 50 ergot alkaloids have been isolated and identified [3,4], and once alkaloids are synthesized, they can convert back and forth between the toxic *R* epimer and the potentially less harmful *S* form [5]. Factors causing conversion among epimers are, as yet, poorly defined, although both pH and matrix effects have been implicated [6]. Another factor complicating the determination of allowable limits for alkaloids in feed is that ergot alkaloids may impact multiple physiological systems [7], potentially interacting with serotoninergic, dopaminergic, and adrenergic receptors [1]. Accordingly, only limited information is available regarding the effects of cereal ergot on growth or reproductive functions in ruminants. In a previous study, we determined that pelleting diets reduced the negative impacts of cereal ergot in growing lambs as compared to feeding mash diets, although the maximum concentration of alkaloids evaluated averaged 433 ppb of total *R* epimers [8]. Consequently, the objectives of this study were to pellet all diets and evaluate a range of dietary alkaloid concentrations, including the maximum allowed for cattle and sheep in Canada, 2 to 3 ppm [9]. Diets were evaluated for both *R* and *S* epimers, and impacts were observed on animal performance, metabolism of nutrients, carcass characteristics, and recovery of individual alkaloids in feces.

## 2. Results

### 2.1. Dietary Ergot Alkaloid Concentrations

Total alkaloids (*R* + *S* epimers) in diets were: Control, 34; 930; 1402 and 2447 ppb, respectively (Table 1). Limits of detection (LOD) using this methodology ranged from 0.1 to 4 ppb for the majority of alkaloids, with the exception of ergosine, where LOD was 0.9 to 19 ppb [10]. Ergocristine was the dominant *R* epimer followed by ergometrine, with ergocristinine the dominant *S* epimer in all diets.

### 2.2. Effects of Alkaloid Concentration on Nutrient Digestibility and Recovery of Alkaloids in Feces

Nutrient digestibility was not impacted by alkaloid concentration (*p* ≥ 0.20; Table 2). Similarly, dietary treatment did not affect any measure of nitrogen metabolism (Table 3). Measures of nitrogen metabolism were affected by period (Table 3). Recoveries of the majority of alkaloids in feces decreased with increasing dietary alkaloid concentration (Table 4), with the exception of ergometrine and its *S* isomer, which increased (*p* < 0.01) with increasing alkaloid concentration. For all alkaloids, with the exception of ergocornine and ergocryptine, recovery varied by period (*p* < 0.05). Ergocorninine was consistently the least recoverable alkaloid (average of 7.3 ± 0.2%) in feces, while ergometrine and ergotaminine were the most (average of 25.9 ± 0.6% and 26.1 ± 1.1%, respectively, in treatments with added alkaloids).

### 2.3. Effects of Ergot Alkaloids on Growth Performance and Carcass Characteristics of Lambs

Lambs received experimental diets for 42 to 91 days. Initial and final body weight (BW), dry matter intake (DMI) and feed efficiency did not differ among treatments (*p* ≥ 0.18; Table 5). Increasing alkaloid concentration caused a linear decrease in average daily gain (ADG; *p* < 0.01), with ADG being lower (*p* < 0.01) for lambs consuming 2447 ppb alkaloids as compared to all other diets. Compared to control lambs, rectal temperatures were 0.33 °C higher (*p* < 0.01) in lambs fed diets with added alkaloids. Similarly, serum prolactin concentrations linearly declined with increasing alkaloid concentration (*p* < 0.01; Table 5). Clinical symptoms associated with alkaloid toxicosis were not evident, as lameness or tissue necrosis was not observed, although some lambs receiving diets with alkaloids were abnormally calm during handling. No mortalities occurred during the experiment.

Alkaloid dosage had no impact on hot carcass weight (*p* = 0.45) or grade rule measurement (*p* = 0.49; Table 5). However, dressing percentage was lower for lambs receiving 1402 ppb alkaloids as compared to those receiving Control and 2447 ppb diets (*p* = 0.02). No livers or carcasses were condemned at the commercial abattoir, and no abnormalities in fat cover were observed.

## 3. Discussion

### 3.1. Alkaloid Profiles

Similar to previous studies of cereal ergot [4,11], ergocristine was the most prevalent *R*, and ergocristinine the most prevalent *S* epimer across all diets. Ergometrine, an alkaloid commonly used to stimulate uterine contractions in humans [12], was the second-most predominant alkaloid, with ergotamine third or fourth in prevalence. Alkaloid profiles in the present study differed from those of Coufal-Majewski et al. [8], as different sources of ergot-contaminated screenings were used in each study. In our previous study, ergocristine accounted for > 50% of *R* epimers, and was approximately 3 times greater in concentration than the next-most concentrated alkaloid, ergocryptine. In the present study, ergocristine was approximately equal in concentration to ergometrine for 930 and 1402 ppb diets and was 1.8 times the concentration of ergometrine in the 2447 ppb diet. Consequently, 1402 and 2447 ppb diets in the present study were the only diets to have concentrations of ergocristine exceeding that previously evaluated by Coufal-Majewski et al. [8]. Potentially, the concentration of ergocristine in the 930 ppb diet was insufficient to produce negative impacts, even though total *R* epimers in the 930 ppb diet were 1.6 times that evaluated in the previous study.

The importance of relative concentrations of individual alkaloids as compared to absolute concentration of total alkaloids is poorly understood. Above a certain threshold, the liver can no longer detoxify alkaloids, leading to clinical symptoms such as tissue necrosis or death [13], although both concentration of total alkaloids and their proportion to each other will influence the negative effects observed [14]. Ergot alkaloids are thought to have additive effects [15], but Dänicke and Diers [14] found evidence of interactions among individual alkaloids in studies of liver function in piglets, where a diet with a lower total concentration had enhanced toxicity compared to a diet with a higher total alkaloid concentration. The reverse may also possibly be true, where certain relatively abundant alkaloids may modulate overall impacts by blocking metabolism of other, potentially more toxic alkaloids.

Ergocristine, ergocornine, ergocryptine and ergotamine all cause blood vessels to contract, with ergotamine eliciting the most potent response in terms of intensity and duration [16]. Approximately 10 molecules of ergocristine or ergocornine would equal the vasoconstrictive potential of ergotamine [17]. Ergotamine and ergocryptine exhibit similar binding properties to D2-dopamine receptors in cell culture, with their affinity being greater than ergometrine [18]. Ergoamide derivatives such as ergometrine mainly promote psychotropic effects, while vasomotor effects and the reduction in serum prolactin concentration are less pronounced [15]. In the present study, as ergocristine was approximately 2.5 times the concentration of ergotamine or ergocryptine; it is likely that ergocristine was responsible for the majority of the reduced growth performance. However, due to the complexity of evaluating interactions among ergot alkaloids, Dänicke [1] suggested reporting animal responses to alkaloids solely on the basis of total dietary ergot alkaloids. Alternatively, Craig et al. [17] has suggested conversion of alkaloids to “ergotamine equivalents” based on the vascular potency of individual alkaloids, although ergotamine equivalents for ergometrine and ergosine would need to be determined before this technique could be applied to cereal ergot.

### 3.2. Alkaloids and Their Epimers

Ergot alkaloids are relatively stable; however, epimerization can be induced with high or low pH, different solvents, and upon exposure to strong light [19]. Consequently, storage conditions (best below −20 °C), handling and analysis need to be consistent to avoid altering alkaloid epimerization [6]. While ergot alkaloids produce a positive charge in acid solutions and are neutral in alkali [19], the actual mechanisms leading to conversion between epimeric forms are unknown [20]. In addition, epimerization can be bi-directional in ergopeptines (i.e., ergometrine, ergotamine, ergosine, ergocristine, ergocryptine and ergocornine), either forming toxic C8-(*R*) isomers or more inert C8-(*S*) isomers referred to as ergopeptinines [21], although *S* epimers may also produce toxic effects [22], and the toxicity of *S* epimers requires additional investigation. Similar to the study of Mainka et al. [23], which evaluated contaminated rye, concentration of *S* epimers in our study averaged approximately 1/3 that of *R* epimers across all diets (Table 1). However, the ratio of *R***/***S* epimers did vary among alkaloids. For example, the *S* epimer only accounted for 1% of ergometrine, suggesting that further conversion of ergometrinine to ergometrine was unlikely. In contrast, if conversion of the *S* epimer to the *R* epimer was favored for ergocorninine or ergocristinine, the concentration of *R* epimers of these alkaloids could increase by an average of 1.7 times. Further investigation of the factors that promote conversions among epimers is necessary to determine if allowable limits should be defined as total alkaloids (*R* + *S* epimers), or solely as the *R* epimers in feed.

### 3.3. Nutrient Digestibility and Alkaloid Recovery in Feces

Microbial digestion may enhance ruminal ergot alkaloid solubility through the metabolism of ergovaline alkaloids to lysergic acid [24]. However, it is unknown whether alkaloid solubility is strictly a function of particle size reduction, or a function of conversion from low-soluble alkaloid species to alkaloids with greater solubility. Moyer et al. [25] found that less than 50% of ergovaline was soluble in in vitro ruminal fermentations, with almost 100% of the soluble components being transformed into an ergot alkaloid derivative within 46 h. Schumann et al. [26] determined that approximately 30% of cereal ergot alkaloids were metabolized prior to the duodenum in dairy cows, although the toxicity of alkaloid metabolites produced in the rumen and elsewhere in the digestive tract requires additional study.

The current study used a 2-week adaption period between each digestibility collection period to reduce possible carry-over effects of alkaloids. Others have observed that alkaloids were undetectable in urine within 96 h after steers were switched from grazing endophyte-infected tall fescue to endophyte-free tall fescue [27]. Acknowledging that alkaloids have the ability to suppress prolactin production, Aiken et al. [28] found that prolactin returned to pre-exposure concentrations 3 d after the removal of ergot-contaminated feed. Schumann et al. [26] used a 2-week adaptation period to cereal ergot alkaloid-contaminated diets before collection of feces and urine. These studies suggest that the clearance phase chosen (2 weeks) was adequate for lambs to avoid carry-over of alkaloids although residence time of alkaloids in tissues is a current subject of study [29].

Increasing ergot content in feed has reduced nitrogen (N) intake and digestibility in livestock fed endophyte-infected hay [30] and in pigs fed ergot-contaminated rye [23,31]. Many of these observations are likely associated with a reduction in feed intake [32]. In the current study and that of Coufal-Majewski et al. [8], ergot alkaloid concentrations did not impact any measure of nitrogen metabolism, and there were no reductions in feed intake. Some measures of nitrogen digestibility were affected by period, likely due to increasing N intake with growth of the lambs. Concentrations of ergot alkaloids 1.7 times greater than the highest concentration fed to lambs in our study were required to reduce N digestibility in pigs [23], although allowable concentrations for ergot alkaloids in diets for swine are double those of sheep or cattle (from 4 to 6 ppm) [9]. Consequently, greater concentrations of alkaloids, or an alkaloid profile that promotes a reduction in feed intake by lambs, may be necessary before reduced N metabolism is observed. It is important to note that the most adverse effects of ergot alkaloids are typically observed during periods of heat stress [33] and cold stress [34], conditions that were not encountered in the present study.

Between 14% and 18% of total alkaloids were recovered in feces of lambs receiving diets containing added alkaloids. Although some alkaloids would be excreted in urine, alkaloid recoveries in the present study were similar to the approximately 24% of ingested cereal alkaloids recovered from the feces of dairy cows [26]. Ergot alkaloids were not analyzed in urine in the present study, as the urine was acidified (pH 2) to prevent loss of ammonia, and the low pH would have influenced epimerization and analysis of the alkaloids [6]. Recovery of ergometrine and ergometrinine in feces increased with increasing dietary concentrations, possibly as proportionally less of these alkaloids were excreted in urine [27]. In contrast to ergometrine and ergometrinine, concentrations of other alkaloids in feces either remained constant, or tended to decrease with increasing dietary concentrations.

All alkaloids in the present study had similar ratios of recovery of *R* and *S* epimers, although Dänicke [29] found greater recoveries of *R* than *S* epimers in the excreta of chickens. Mechanisms for metabolism of ergot alkaloids likely differ across livestock species [35]. Accordingly, alkaloid concentrations of diets fed to chickens in the study of Dänicke [29] were up to 7-fold greater than those of the current study. Individual animals of the same species are also known to differ in their tolerance to ergot alkaloids [7]. In the present study, with the exception of ergocornine and ergocryptine, recovery of alkaloids in feces significantly varied with period, suggesting that the metabolism of other alkaloids may have differed among individual lambs. Excretion of alkaloids in feces also increased during each subsequent period of the digestibility study, suggesting that lambs may have adapted to alkaloids over time through increasingly efficient metabolic pathways [27]. Similarly, Dänicke and Diers [14] observed that adverse effects of cereal ergot exposure in piglets became less as the experimental period progressed, likely due to metabolic adaptation to alkaloids.

Metabolic responses to dietary ergot are undoubtedly complex, as NDF digestibility by lambs decreased at 433 ppb *R* epimers [8], with no impacts of dietary treatments on fiber digestibility noted in the current study. The source of contaminated screenings and alkaloid profiles differed between studies, demonstrating the difficulty of determining the concentration of ergot alkaloids where no adverse effects are observed. In the study of Coufal-Majewski et al. [8], the maximum concentration at which no adverse effects were noted for nutrient digestibility of pelleted diets was 185 ppb *R* epimers, as compared to 1790 ppb *R* epimers in the present study. Dänicke [1] noted the difficulty in predicting the net effect of an ergot alkaloid mixture due to dose- and proportion-associated interactive effects. Further investigation to determine and evaluate the most common alkaloid profiles present in a region or country could be useful in determining the lower limit at which that profile has a negative impact on digestibility, growth performance, and animal health.

### 3.4. Effects of Ergot Alkaloid Concentrations on Growth Performance

Behavior of some lambs receiving diets with added alkaloids was judged to be unusual by barn staff (abnormal calm while being herded to the weigh crate), potentially due to the psychotropic effects of ergometrine [15]. Alterations in behavior would have had limited impact on performance of individually penned sheep with no need to flee from predators and readily accessible ad libitum feed and water. Although not quantified in the present study, impacts of ergot alkaloids on animal behavior are an important consideration due to the potential for reduced animal fitness, especially under commercial management conditions.

Koontz [32] noted that ruminal volatile fatty acid profile was altered by alkaloid ingestion, which could cause a reduction in nutrient absorption due to reduced feed intake. In contrast, the alkaloid concentration of diets did not impact DMI in the current study or that of Coufal-Majewski [8], providing evidence that secondary effects of alkaloid ingestion may have a greater impact on lamb performance than alterations in feed intake. A reduction in feed intake has been identified as the most sensitive endpoint for determining the adverse effect concentration of ergot alkaloids in broiler chickens [1], but based on the results of the present study, adverse effects in lambs are likely to occur before a reduction in feed intake would be noted.

Coufal-Majewski et al. [8] observed only a minor reduction in the ADG in lambs receiving diets containing 433 ppb *R* epimers; whereas in the present study, ADG was reduced by 27% as compared to Controls in lambs fed 1790 ppb *R* epimers. All diets in the present study were pelleted, with alkaloids measured after pelleting, which may have helped to moderate alkaloid impacts on performance, as reductions in ADG in the study of Coufal-Majewski et al. [8] were more pronounced with mash diets. However, different sources of ergot with varying alkaloid profiles makes comparison of the two studies difficult. Additionally, alkaloid analyses in the two studies were performed by different labs to facilitate analysis of both *R* and *S* epimers in the present study. As 2447 ppb is within the maximum allowable alkaloid range in diets for sheep and cattle in Canada (from 2 to 3 ppm) [9], it was expected that a diet with this concentration of alkaloids would impact performance of the lambs, although current Canadian regulations do not distinguish between *R* and *S* epimers. More surprising was the lack of impact of the 930 and 1402 ppb diets on ADG, as lower concentrations of *R* epimers in a previous study led to a significant reduction in ADG [8].

Ergot alkaloids are known to impact lipid metabolism in sheep and other livestock, with increasing concentrations causing observable changes in fat cover and liver health [36,37]. In the current study, carcass dressing percentage was reduced for lambs fed the 1402 ppb diet compared to other treatments, possibly because of differing proportions of alkaloids, as relative concentrations of ergocornine and ergocryptine were elevated for this diet. Similarly, nutrient digestibility measures in a separate group of lambs tended to be depressed for lambs receiving the 1402 as compared to the 2447 ppb diet (Table 2 and Table 3). Although all diets were prepared from the same source of contaminated screenings, due to variations in alkaloid concentrations and profiles in individual ergot bodies [1], it was not possible to achieve identical alkaloid profiles across diets.

Additional study is required to evaluate impacts of individual or combinations of alkaloids on lipid metabolism as effects of ergot-contaminated feed on livestock have been inconsistent [14]. Lambs fed all diets contaminated with ergot had dressing percentages <48%, below the range for lambs to receive maximum market value at the abattoir (from 48 to 57.9%) [38]. This would cause a reduction in overall revenue from feeding ergot-contaminated diets, in addition to that of the reduced ADG noted for the 2447 ppb diet

### 3.5. Effects of Ergot on Serum Prolactin and Rectal Temperature

Ergot alkaloids can act as either an agonist or antagonist for physiological functions depending on what type of alkaloid is present, frequently affecting the dopamine receptor [39]. Administration of ergocristine, a dopamine agonist, can instantaneously block release of prolactin [39,40]. Ergocristine was the most prevalent alkaloid in feed in the present study, which may explain the significant linear reduction in serum prolactin concentration with increasing alkaloid concentration. Similarly, Coufal-Majewski et al. [8] observed a decline in serum prolactin concentrations of lambs even at dietary alkaloid concentrations as low as 170 ppb. Rams grazing endophyte-infected pastures have also exhibited reduced serum prolactin concentrations and testicle weight, but the fertility of rams was unaffected one year after exposure to alkaloids [41].

Rectal temperatures recorded for control lambs were within a normal range (38.5 to 39.9 °C) [42], but lambs receiving diets with added alkaloids exhibited slightly greater rectal temperatures. Gadberry et al. [43] found that dietary ergot alkaloids increased susceptibility of sheep to heat stress. However, to induce heat stress, ambient temperatures typically need to exceed 31 °C [30,44]. As the average daily ambient temperature never exceeded 27.4 °C in the current study, heat stress was not induced. Future studies are required to address impacts of temperatures above or below the thermo-neutral zone on toxicity of ergot alkaloids.

### 3.6. Developing Allowable Limits for Ergot Alkaloids Based on These Data

In the present study, symptoms of alkaloid toxicosis were demonstrated in lambs receiving ergot-contaminated diets, although in the case of carcass dressing percentage, negative impacts of alkaloids were most pronounced for lambs receiving the 1402 ppb diet. Serum prolactin and ADG linearly decreased with increasing dietary alkaloids, but ADG was reduced in lambs fed 2447 ppb as compared to 1402 ppb ergot alkaloids. Concentrations of alkaloids present in diets appear to be poor predictors of animal performance as diets containing 433 ppb total *R* epimers caused significant reduction in ADG of lambs [8], while a concentration of 1790 ppb *R* epimers was required in the present study to significantly reduce ADG. Based on these results, it will be challenging to develop consistent, scientifically justifiable allowable limits for cereal ergot alkaloids in livestock feed.

## 4. Materials and Methods

All experiments were conducted between June and September 2016 at the Lethbridge Research Centre (LRC) of Agriculture and Agri-Food Canada, Protocl #1507 was approved 25 February 2015 for this project by the LRC Animal Care Committee according to the guidelines of the Canadian Council on Animal Care [45].

### 4.1. Ergot Source and Alkaloid Determination

A 1.0-kg representative sample of barley screenings from a single bag obtained from the Canadian Feed Research Centre, North Battleford, SK was analyzed for thirteen ergot alkaloids (chanoclavine, ergocornine, ergocorninine, ergocristine, ergocristinine, ergocryptine, ergocryptinine, ergometrine, ergometrinine, ergosine, ergosinine, ergotamine and ergotaminine) by Biomin Research Center (Tulln, Austria). Alkaloids were determined by HPLC-MS/MS [6], based on retention time and intensity ratio as compared to verified standards. In brief, a 500 g sample of feed or feces was subsampled (5.0 g), ground (through a 1-mm screen) and stored in 20 mL of extraction solvent (acetonitrile/water/acetic acid 79:20:1, *v*/*v*/*v*) overnight in darkness at room temperature, to avoid alkaloid degradation and allow equilibrium to establish between analytes and matrix [6]. Samples were then extracted for 90 min and centrifuged for 2 min at 1500× *g*, with 350 μL aliquots diluted with solvent (acetonitrile/water/acetic acid 79:20:1, *v*/*v*/*v*). Following mixing, 5 μL of extracted alkaloids (in solution) was injected into an Agilent 1290 HPLC (Aligent Technologies Inc., Waldbronn, Germany) combined with a 5500 QTrap mass spectrometer (Applied Biosystems, Vienna, Austria), in the selected reaction monitoring mode incorporating electrospray ionization monitoring. Validation of the method was performed according to SANCO criteria [6] with repeatability based on analysis of 5 replicates considered acceptable if residual standard deviation <20% for all alkaloids.

### 4.2. Treatments and Diet Preparation

To ensure uniform distribution of ergot in diets, a Wiley^®^ Cutting Mill (Thomas Scientific, Waltham, MA, USA) was used to grind contaminated screenings through a 1-mm screen. Screenings were analyzed three times for total alkaloids as described previously. Based on their mean total alkaloid concentration, ground screenings were then substituted for barley in diets containing added alkaloids. To make the diets, pelleted alfalfa was first milled. Four pelleted diets were then made and analyzed for total alkaloids each in a single batch (2500 kg; Table 6) and used for both growth and digestibility studies. Diets were Control (no added alkaloids), 930, 1402 and 2447 ppb total alkaloids based on *R* and *S* epimers combined (Table 1), with alkaloid concentrations measured after the diets were pelleted.

### 4.3. Nutrient Digestion and Metabolism of Lambs

Eight newly weaned Canadian Arcott × Rideau Arcott ram lambs were selected based on similar initial BW (30.0 ± 3.08 kg), to determine the digestibility of diets using a replicated Latin square design. Lambs had not previously been exposed to dietary ergot. Each period lasted for 21 d, with 14 d for adaption and the final 7 d for sample collection. Lambs were randomly assigned to diet, fed once daily, and restricted to 95% of ad libitum intake during the week of total collection. Orts were collected and weighed daily during the adaption period to determine DMI and the health of lambs was visually monitored daily. Seven d prior to the first collection period, lambs were shorn and pre-fitted with strap-on canvas fecal collection bags. Lambs were individually fed in crates with transparent panels to allow observation of other lambs and to minimize stress during the collection period.

Feces and urine were collected daily throughout the last 4 d of each period. Sulfuric acid (4 M, 100 mL) was placed in urine collection buckets each morning to prevent ammonia volatilization. Fecal collection bags were emptied once daily at 800 h. Feces that fell into the crate were incorporated in calculations of total fecal production, but not included in the subsample. Subsamples of feces (15%) and urine (10%) were collected on each collection day, with a second fecal subsample used to calculate daily fecal dry matter (DM). Subsampled urine was pooled, mixed and further divided into two 80 mL urine containers and stored at −10 °C prior to analysis.

### 4.4. Laboratory Analyses

As a measure of bioavailability of ergot alkaloids, feces were collected to determine percentage recovery of alkaloids. Pooled feces were thawed and dried at 55 °C prior to being ground. Both fecal and diet samples were ground through a 1-mm screen (Cutting Mill SM 100; Retsch Inc., Newtown, PA, USA). Samples were prepared and analyzed for analytical DM, organic matter (OM), crude protein, neutral detergent fiber (NDF), acid detergent fiber (ADF) and crude fat content as described previously [8]. Briefly, diet and fecal samples were further dried (105 °C for 24 h) to estimate analytical DM; ashed at 550 °C for 5 h to estimate OM and ball ground (Mixer Mill MM200; Retsch Inc., Newtown, PA, USA) prior to analysis for N (Method #990.03) [46] using a 2100 Elemental Combustion Analyzer (Carlo Erba Instruments, Milan, Italy). Urine was thawed prior to analysis, diluted (1:5; urine:water), and 50 μL of the sample was pipetted into sample cups prior to drying overnight at 55 °C. Known concentrations of finely-ground hard red spring wheat were used to develop a standard curve for N analysis. The protocol of Van Soest et al. [47] using an Ankom 200 fiber analyzer (Ankom Technology, Macedon, NY, USA) expressed NDF inclusive of residual ash (with the addition of amylase and sodium sulfite; aNDF) and ADF analyzed following the AOAC method 973.18 [46] exclusive of residual ash (ADFom). Crude fat content of the samples was determined using ether extraction following AOAC method 920.39 [46]. Composite fecal samples (approximately 400 g) from the two animals in the digestibility trial fed each diet in each period were ground through a 1-mm screen and analyzed for alkaloid *R* and *S* epimers: (ergocornine, ergocorninine, ergocristine, ergocristinine, ergocryptine, ergocryptinine, ergometrine, ergometrinine, ergosine, ergosinine, ergotamine, ergotaminine) and chanoclavine [6].

### 4.5. Performance Study

Forty-seven newly weaned Canadian Arcott × Rideau Arcott ram lambs (30 ± 5.8 kg), were blocked by weight and then randomly assigned to one of four diets and housed in individual pens bedded with wood chips from June to September 2016. A total of 48 lambs started the performance study, but one lamb was moved to the digestibility trial after the first period to replace a lamb with chronic diarrhea. Lambs were not previously exposed to dietary ergot and had ad libitum access to diets (Table 6) and water throughout the experiment. Feed deliveries were recorded daily, with daily refusals collected and weighed for determination of daily DMI. Individual BW was recorded weekly and ADG was determined by dividing weight gain (initial BW–final BW) by the number of days in the study. Feed efficiency was calculated as the ratio between ADG and DMI (g of BW gain/g DMI). Lamb health was monitored daily by barn staff, and ambient temperature in the barn was recorded three times daily throughout the experiment. Rectal temperature of all lambs was recorded at week 10 of the performance study using a digital thermometer (GLA M750, GLA Agricultural Electronics, San Luis Obispo, CA, USA).

Blood samples were collected from 36 lambs prior to commencing experimental diets; after reaching 35 kg BW, and at slaughter weight (≥45 kg). Serum was obtained by centrifugation (2000× *g* for 15 min at 4 °C) and stored in a 1 mL screw-cap tubes in duplicate at ‒80 °C. Prolactin concentrations were determined in serum using a double antibody radioimmunoassay with ovine prolactin (100:1; first antibody) and rabbit globulins (500:1; second antibody) and reagents obtained from the National Hormone and Pituitary Program (Harbor-UCLA Med Ctr, Torrance, CA, USA) as outlined by Coufal-Majewski et al. [8].

Lambs were removed from the experiment upon reaching ≥45 kg and slaughtered at a commercial abattoir (*n* = 38; SunGold Meats Ltd., Innisfail, AB, Canada). Carcass traits were assessed according to Canadian Food Inspection Agency standards [48].

### 4.6. Statistical Analyses

For the performance study, DMI, ADG and feed efficiency were analyzed in a completely randomized design by 2-way ANOVA using the MIXED procedure of SAS, with alkaloid concentration as a fixed effect and week as a repeated measure (SAS Inst. Inc., Cary, NC, USA). The minimum values of AIC (Akaike’s Information Criterion) were used to select the covariance structure which were estimated as N * In (error-sum-or-squares/N) + 2 * (number of independent variable parameters including the intercept term), where N = the number of observations. Prolactin data collected over the three sampling points were normalized by log transformation prior to analyses. Initial BW, final BW and carcass data were analyzed using the MIXED procedure. Digestibility and nitrogen metabolism calculations were also analyzed using the MIXED procedure in a crossover design with period as fixed and lamb within dietary treatment as random effects. Significance was acknowledged for both studies at *p* ≤ 0.05. Linear and quadratic effects were evaluated by using planned orthogonal polynomial coefficients for each parameter when Type 3 tests of fixed effects ≤0.05. Only linear responses to increasing concentrations of alkaloids were reported in the text as quadratic contrasts were not significant.

## Figures and Tables

**Table 1:** Concentrations (ppb) of alkaloid *R* and *S* epimers in experimental diets fed to lambs.

	Diet							
	Control ^1^		930 ppb		1402 ppb		2447 ppb	
Alkaloid	*R* epimer	*S* epimer	*R* epimer	*S* epimer	*R* epimer	*S* epimer	*R* epimer	*S* epimer
Chanoclavine	2	NA^2^	4	NA	5	NA	6	NA
Ergocornine/inine	1	1	60	51	100	73	125	105
Ergocristine/inine	12	7	219	112	338	169	704	361
Ergocryptine/inine	1	2	90	26	148	32	216	51
Ergometrine/inine	ND ^3^	2	209	3	273	3	382	4
Ergosine/inine	ND	ND	29	22	38	30	82	61
Ergotamine/inine	7	1	85	22	152	42	275	76
Total epimer	22	12	695	235	1054	348	1790	657
Total alkaloids	34		930		1402		2447	

^1^ No added alkaloids; ^2^ NA, not measured; ^3^ ND, not detected.

**Table 2:** Nutrient digestion in lambs (*n* = 8) fed experimental diets.

	Diet				Probability		
Digestibility, %	Control ^1^	930 ppb	1402 ppb	2447 ppb	SEM ^2^	Dose	Period ^3^
Dry matter	71.5	72.2	70.1	71.9	0.80	0.29	0.46
Organic matter	72.9	73.3	71.5	73.3	0.80	0.37	0.60
Crude protein	73.7	75.3	72.8	73.1	1.21	0.49	0.86
Neutral detergent fiber	38.4	39.7	34.5	39.4	1.89	0.20	0.46
Acid detergent fiber	23.9	25.7	21.9	25.7	1.81	0.40	0.51

^1^ No added alkaloids; ^2^ SEM: Standard error of mean; ^3^ Overall effects of period of digestibility study (*n* = 4).

**Table 3:** Urinary nitrogen consumption, excretion and retention by ram lambs (*n* = 8) in the digestibility study.

	Diet				Probability		
Parameter	Control ^1^	930 ppb	1402 ppb	2447 ppb	SEM ^2^	Dose	Period ^3^
Crude protein digestibility %	73.7	75.3	72.8	73.1	1.21	0.49	0.86
N intake, g	42.9	45.5	45.0	46.8	1.66	0.45	<0.01
N digested, g	31.0	33.7	32.0	33.6	1.11	0.31	<0.01
N retained, g	13.3	14.4	13.7	14.4	0.80	0.75	0.07
N output, g	18.4	20.2	18.7	20.5	0.91	0.32	<0.01
% of N retained (intake)	31.5	33.0	31.3	32.1	1.53	0.86	<0.01
% of N retained (digested)	43.7	44.2	43.8	44.4	2.06	0.99	<0.01

^1^ No added alkaloids; ^2^ SEM: Standard error of mean; ^3^ Overall effects of period of digestibility study (*n* = 4).

**Table 4:** Recovery of individual alkaloids in feces of digestibility study lambs (*n* = 8).

	Recovery, %				Probability		
Alkaloid	Control ^1^	930 ppb	1402 ppb	2447 ppb	SEM	Dose	Period ^2^
Chanoclavine	16.5	10.5	11.2	9.1	0.3	<0.0001	0.04
Ergocornine	0.0	10.8	11.2	10.8	0.2	<0.0001	0.13
Ergocorninine	13.9	6.5	7.4	7.4	0.2	<0.0001	0.02
Ergocristine	49.4	17.4	18.7	11.7	0.7	<0.0001	0.002
Ergocristinine	33.5	17.3	16.8	11.8	0.5	<0.0001	0.04
Ergocryptine	ND	9.2	10.6	9.6	0.6	<0.0001	0.14
Ergocryptinine	18.4	12.1	15.1	12.3	0.3	<0.0001	0.04
Ergometrine	ND	21.6	26.7	29.5	0.6	<0.0001	0.02
Ergometrinine	9.9	15.6	17.8	17.9	0.3	<0.0001	0.03
Ergosine	ND	21.8	27.0	18.1	0.6	<0.0001	0.03
Ergosinine	ND	14.5	19.1	11.8	0.4	<0.0001	0.02
Ergotamine	44.5	28.2	28.2	18.7	0.9	<0.0001	0.001
Ergotaminine	ND	31.9	25.6	20.8	1.1	<0.0001	0.005

ND: Not Detected; ^1^ Control, no added alkaloids; ^2^ Period, overall effects of period of digestibility study (*n* = 4).

**Table 5:** Growth performance, rectal temperature (°C) and carcass characteristics of lambs receiving experimental diets for 6 to 13 weeks.

	Diet				Probability	
Parameter	Control ^1^	930 ppb	1402 ppb	2447 ppb	SEM	Dose
Growth (*n* = 47)						
Initial body weight (kg)	28.7	29.9	30.3	31.3	1.51	0.72
Final body weight (kg)	50.5	50.9	50.8	50.3	1.01	0.95
Dry matter intake (g/d)	1500.6	1469.4	1512.1	1406.0	115.4	0.91
Average daily gain (g/d)	353.6a	314.3a	339.4a	256.9b	16.07	<0.01
Feed conversion (gain:feed)	0.32	0.24	0.26	0.24	0.10	0.18
Rectal temperature ^2^	39.8a	40.0b	40.3b	40.1b	0.09	<0.01
Ln Prolactin (μg/L) ^3^	4.3a	3.4b	3.5b	3.0b	0.23	<0.01
Carcass characteristics (*n* = 38)						
Hot carcass weight (kg)	24.3	23.7	22.7	23.8	0.69	0.45
Dressing percentage	48.2a	46.2ab	44.2b	47.1a	0.87	0.02
Grade rule (mm)	17.1	16.7	15.3	15.5	0.95	0.49

a,b Means within rows with different letters differ (*p* < 0.05). ^1^ Control, no added alkaloids. ^2^ Rectal temperature recorded on d 72. ^3^ Mean prolactin (μg/L) in performance lambs (*n* = 36) from serum samples collected at three stages during the study: (1) at the commencement; (2) Live weight 35 kg; (3) market weight (≥45 kg).

**Table 6:** Diet composition (as fed).

	Diets			
Ingredients, %	Control ^1^	930 ppb	1402 ppb	2447 ppb
Barley grain	53.9	53.8	53.9	53.7
Alfalfa pellets (18% CP)	27.1	27.0	27.0	27.0
Canola meal	16. 8	16.8	16.8	16.8
Canola oil	1.0	1.0	1.0	1.0
Sheep Mineral ^2^	1.0	1.0	1.0	1.0
Limestone	0.3	0.3	0.3	0.3
Vitamin ADE ^3^	0.02	0.02	0.02	0.02
Deccox ^4^	0.013	0.013	0.013	0.013
Ergot screening	0	0.09	0.15	0.23
Dry matter and chemical composition (analytical DM basis)				
Dry matter, %	90.8 ± 2.9	91.0 ± 3.2	90.7 ± 4.0	90.8 ± 3.3
Organic matter	92.2 ± 0.3	92.5 ± 0.4	92.4 ± 0.5	92.9 ± 0.5
Crude protein	18.3 ± 3.8	18.8 ± 4.3	19.0 ± 4.1	19.2 ± 3.9
Neutral detergent fiber	23.9 ± 1.3	25.0 ± 2.0	23.7 ± 5.3	26.8 ± 3.7
Acid detergent fiber	12.7 ± 0.4	12.8 ± 3.9	12.5 ± 4.0	13.0 ± 6.5
Ether extract	3.3 ± 8.9	3.6 ± 4.1	3.8 ± 4.0	3.5 ± 7.2

^1^ no added alkaloids. ^2^ Sheep mineral constituents (%): salt 92.6, potassium magnesium sulfate 4.979, zinc sulfate 0.921, magnesium sulfate 0.835, organic iodine 0.014, 1% selenium premix 0.143, cobalt carbonate 0.004, canola oil 0.398. ^3^ Vitamin ADE constituants: vitamin A 10,000,000 IU, vitamin D 1,000,000 IU, vitamin E 10,000 IU/kg. ^4^ Deccox active ingredient: decoquinate (6%; Zoetis, Kirkland, QC, Canada).
